# Portuguese crypto-Jews: the genetic heritage of a complex history

**DOI:** 10.3389/fgene.2015.00012

**Published:** 2015-02-02

**Authors:** Inês Nogueiro, João C. Teixeira, António Amorim, Leonor Gusmão, Luis Alvarez

**Affiliations:** ^1^Institute of Molecular Pathology and Immunology of the University of PortoPorto, Portugal; ^2^Faculty of Sciences, University of PortoPorto, Portugal; ^3^Instituto de Investigaç ao e Inovaç ao em Saúde, Universidade do PortoPorto, Portugal; ^4^Department of Evolutionary Genetics, Max Planck Institute for Evolutionary AnthropologyLeipzig, Germany; ^5^DNA Diagnostic Laboratory, State University of Rio de JaneiroRio de Janeiro, Brazil

**Keywords:** crypto-Jews, Y chromosome, mtDNA, haplogroups, Portugal

## Abstract

The first documents mentioning Jewish people in Iberia are from the Visigothic period. It was also in this period that the first documented anti-Judaic persecution took place. Other episodes of persecution would happen again and again during the long troubled history of the Jewish people in Iberia and culminated with the Decrees of Expulsion and the establishment of the Inquisition: some Jews converted to Catholicism while others resisted and were forcedly baptized, becoming the first Iberian Crypto-Jews. In the 18th century the official discrimination and persecution carried out by the Inquisition ended and several Jewish communities emerged in Portugal. From a populational genetics point of view, the worldwide Diaspora of contemporary Jewish communities has been intensely studied. Nevertheless, very little information is available concerning Sephardic and Iberian Crypto-Jewish descendants. Data from the Iberian Peninsula, the original geographic source of Sephardic Jews, is limited to two populations in Portugal, Belmonte, and Bragança district, and the Chueta community from Mallorca. Belmonte was the first Jewish community studied for uniparental markers. The construction of a reference model for the history of the Portuguese Jewish communities, in which the genetic and classical historical data interplay dynamically, is still ongoing. Recently an enlarged sample covering a wide region in the Northeast Portugal was undertaken, allowing the genetic profiling of male and female lineages. A Jewish specific shared female lineage (HV0b) was detected between the community of Belmonte and Bragança. In contrast to what was previously described as a hallmark of the Portuguese Jews, an unexpectedly high polymorphism of lineages was found in Bragança, showing a surprising resistance to the erosion of genetic diversity typical of small-sized isolate populations, as well as signs of admixture with the Portuguese host population.

## INTRODUCTION

The purpose of this review is to summarize and critically revise the existing genetic data concerning the Portuguese Sephardic Jewish population. In this regard, other Sephardic population studies will be reviewed for contextualization. The historical background of the Sephardic Jews, with an emphasis on the Portuguese history, will be addressed. With the exception of the studies on the *Chuetas*, an isolated Mallorcan community from the Moslem period, 10–13th centuries (Santamaría Arández, 1997), currently available genetic studies on the original population from Iberia are restricted to Portugal, namely to Belmonte municipality and Bragança district (Northeast Portugal).

Until the decree of expulsion and the establishment of the Inquisition in Portugal during the 15th century, the Portuguese Jewish communities had quite a similar history. From the 15th century on, however, most of the Portuguese Jews were either exiled, or assimilated into the general population with the exception of a few Crypto-Jewish communities. The Crypto-Jewish phenomenon is defined as the secret adherence to Judaism while publicly professing another faith. These communities have kept, for more than 500 years, their hidden religious practices and their cultural identity using complex social strategies. Among these communities, special mention is due to Belmonte, a small town in the center of Portugal and also to several small villages and towns in the Bragança district. Uniparental genetic markers were typed in both communities (Adams et al., 2008; Nogueiro et al., 2010, 2014; Teixeira et al., 2011), showing genetic profiles and levels of genetic diversity in accordance with their dissimilar recent history compared to the Portuguese general population.

## A TROUBLED HISTORY

The settlement of Jewish groups in Iberia undoubtedly occurred long time ago, although the exact date of their appearance is still today uncertain. The oldest archeological evidence of the Jewish presence in Iberia known so far, was recently found in the south of Portugal (Silves) with a chronology of 390 CE (http://www.uni-jena.de/en/News/PM120525_Schrifttafel.html).

Written documents mentioning the presence of Jewish communities in Iberia accumulate from the beginning of the Visigothic period onward: in the 4th century CE, the decisions of the Council of Elvira, particularly the interdiction of marriages between Jews and Christians, confirm their complete integration among Iberian communities (Martins, 2006; Wilke, 2009). The decree of expulsion of all Iberian Jews who would not embrace the Christian faith by the Visigothic King Sisebut in 613 CE marks the beginning of Crypto-Judaism and was a harbinger of their subsequent dramatic history in the Iberian Peninsula (Martins, 2006; Wilke, 2009).

A climate of tolerance between Jews, Muslims and Christians marked the Islamic period in Portugal (from 711 to 1249). Jews were at that time a demographically non-negligible minority with a very heterogeneous social status (Martins, 2006). Over the 11 and 12th centuries, a strong influence of Islamic culture was evident in the Iberian Peninsula, particularly in philosophy, geography, astronomy, mathematics and medicine, providing a cultural blossoming often qualified as the golden age of the Sephardic Jews (Maurício, 1971; Pignatelli, 2000; Tavares, 2004).

From the 12th to the end of the 13th century, the geography of Judaism changed significantly with the Christian settlement policies, to which the first Jewish medieval colonies owe their existence (Wilke, 2009). From the beginning of the Portuguese nation in 1143, until the Expulsion Edict in 1496, the successive Portuguese monarchs balanced the predominance of the Jewish social and economic life against the anti-Judaic clerical and popular pressures (Martins, 2006). Contrasting with the favorable measures toward the Jewish population, several opposite restrictive policies were also adopted by the successive Portuguese kings (Paulo, 1985; Azevedo, 1994; Pignatelli, 2000; Martins, 2006). The 14th century marks a dramatic change in this fragile equilibrium, which nevertheless allowed the emergence of Jewish communes in Portugal. The estimates of the size of the Portuguese Jewish population vary between 30 000 to more than 60 000. This number would increase substantially with the arrival of around 100 000 Spanish Jews fleeing from their country (Carvalho, 1999; Martins, 2006).

In 1478 the Inquisition was established in Spain, followed by the Edict of Expulsion by the Catholic Kings. These events would have a strong impact in the future of the Portuguese Jews (Canelo, 1987; Pignatelli, 2000; Martins, 2006). The initial tolerance toward the Spanish exiles and the Portuguese Jews was, however, doomed. The marriage of the Portuguese King Manuel I to the daughter of the Catholic Kings had severe political implications (Canelo, 1987; Pignatelli, 2000; Martins, 2006). Accordingly, in December of 1496 the King signed the Portuguese Edict of Expulsion, ordering the departure of Moors and Jews by October of the following year (Canelo, 1987; Pignatelli, 2000). However, in May of 1497, about 20 000 Jews from all over Portugal, who were preparing for exile, were forcibly baptized. As a result, there were officially no more Jews in Portugal, and instead, a new identity was created: the *new-Christians* or *Conversos* (Martins, 2006). The ambiguous policies adopted toward these communities, where some rulings favored the Jews and others worked against them, highlights their socioeconomic importance. In fact, King Manuel I of Portugal prohibited inquiries regarding the Jewish faith for 20 years, allowing for an accepted crypto-Judaism (Pignatelli, 2000; Martins, 2006).

The Papal Bull establishing the Inquisition in Portugal was issued in 1536 under the reign of King João III. In the 17 and 18th centuries, the inquisitorial processes intensified and as a result, there was a significant exodus of Jewish people to other countries, particularly of manufacturers and the merchant elite (Paulo, 1985).

By the end of the 15th century estimates of the size of the Jewish population were of about 100 000 people, which translates into 10% of all the Portuguese population at that time. The exact number of people who emigrated is not known, however, it is thought that in 1631 the Jewish population was reduced to 10 000 (Carvalho, 1999). Initially, the Portuguese Jews settled in Amsterdam, London, Hamburg, Turkey, some French and Italian cities, and North Africa; from the mid-16th century, they migrated to the Portuguese colonies in Africa, India and Brazil, and later from the several cities of northern Europe to the New World, (e.g., Curacao, Paramaribo, and the USA), where Jewish colonies founded synagogues with the Portuguese rite (Carvalho, 1999; Pignatelli, 2000; Martins, 2006).

The rebirth of the Jewish communities in Portugal took place in the early 19th century, when the Marquis of Pombal ended the official discrimination and persecution performed by the Inquisition (Carvalho, 1999; Martins, 2006). The Israeli Jewish community of Lisbon was founded by Sephardic Jews from North Africa (Pignatelli, 2000; Martins, 2006), and in the 20th century, the communities of Porto, Bragança, Belmonte, Faro and the Azores emerged (Paulo, 1985; Canelo, 1987; Pignatelli, 2000; Martins, 2006).

In particular, the communities of Bragança and Belmonte resurfaced thanks to the work of Samuel Schwartz and Captain Barros Basto, who started a movement in the early 20th century which aimed to bring back the Crypto-Jews to normative Judaism. While in Belmonte the community is still dynamic today, the Bragança community was dissolved in 1934, shortly after its appearance and its population dispersed in the region, though a strong sense of identity among their Jewish descendants is still well alive today.

## THE CRYPTO-JEWS‘ GENETIC HERITAGE

The genetic heritage of Jewish populations has been deeply scrutinized at the population level as well as for the medical implications, using uniparental and autosomal markers (Hammer et al., 2000; Ostrer, 2001; Bauchet et al., 2007; Adams et al., 2008; Behar et al., 2008; Olshen et al., 2008; Kopelman et al., 2009; Elhaik, 2013) and more recently through genome-wide approaches (Seldin et al., 2006; Atzmon et al., 2010; Behar et al., 2010; Campbell et al., 2012; Velez et al., 2012; Ostrer and Skorecki, 2013).

In the medical field, the Ashkenazi community has been by far the most investigated Jewish group. There is a vast list of published studies focusing on genetic diseases in the Ashkenazi population (Ostrer, 2001; Alcalay et al., 2014; Feldman et al., 2014; Tafe et al., 2015) contrasting with the low number of such studies in other Jewish groups, particularly in the Sephardic group. The term “Sephardic” frequently includes not just the original Portuguese/Spanish Jewish populations but also all other Iberian exiled communities that follow the Sephardic rite and it can as well be used as a synonym for non-Ashkenazi groups, oftentimes engulfing the Mizrahi group. Thus, genetic diseases commonly found in Sephardic Jews can comprise particular disorders that are exclusive to a specific sub-populations of this heterogeneous group (Rosner et al., 2009).

With regard to the genetic disorders of the Sephardic Jews who stayed in Iberia after the decrees of expulsion and the establishment of the Inquisition, very little is known. The only recognized Jewish population in the Spanish territory that follows this criterion are the *Chuetas*. Several reports are available with clinical relevance (Buades et al., 1995; Domingo et al., 2000; Guix et al., 2002; Cambra et al., 2009). As to Portugal, there is only one report on a Jewish genetic condition, an autosomal recessive form of retinitis pigmentosa studied in the Crypto-Jews of Belmonte (Gerber et al., 2000). Similar to other Sephardic specific variants, as the consequence of sustained inbreeding practices, this seems to have arisen 200–500 years ago, after the establishment of this isolated population in Belmonte (Gerber et al., 2000). Recent studies also reported a high prevalence of this disorder in Ashkenazi Jews (Zelinger et al., 2011; Zuchner et al., 2011; Venturini et al., 2014) but caused by a mutation in a different gene.

### MONOPARENTAL GENETIC MARKERS

In recent years, the analysis of uniparentally inherited genetic systems, the non-recombining region of Y chromosome (NRY) and mitochondrial DNA (mtDNA), has played a central role in disclosing the demographic events that have shaped modern human population structure (Brown, 1980; Cann et al., 1987; Hammer, 1994; Jobling and Tyler-Smith, 2003). Both genetic systems have been used since the 90 s in the analysis of Iberian Peninsula populations, producing a detailed genetic landscape (Arroyo-Pardo et al., 2007; Adams et al., 2008; Capelli et al., 2009; Santos et al., 2014) and the assessment of the contributions from the various parental populations to the Iberian genetic composition is currently a very active research topic.

### Y CHROMOSOME STUDIES IN SEPHARDIC POPULATIONS

The search for a common Middle Eastern origin of contemporary Jewish male lineages started with Hammer and Skorecki’s landmark genetic study of the Cohanim (Hammer et al., 1997), a priestly lineage of the Jewish religion. In this study they defined the “Cohen Modal Haplotype” or “CMH” and showed a common origin for this lineage among both Sephardim as well as Ashkenazim Jews. Thomas et al. (1998) found that Y chromosomes of present-day Cohanim and Levites (also a priestly lineage) shared a common origin estimated to date about 3000 years before present.

Subsequent studies (Hammer et al., 2000; Thomas et al., 2000; Nebel et al., 2001) have suggested that most Jewish communities have remained quite isolated from neighboring non-Jewish communities during and after the Diaspora and that the communities from Europe, North Africa, and the Middle East descended from a common Middle Eastern ancestral population.

A high-resolution Y chromosome haplotype analysis on unrelated Israeli and Palestinian Moslem Arabs showed a common pool for the male lineages. However, some significant differences were also detected between Jews and Arabs, suggesting a recent divergence of the Arab clade from the common ancestral population (Nebel et al., 2000).

The research on the Jewish Priestly lineages, Levites and Cohanim was again addressed by Behar et al. (2003) showing that paternal ancestries of Ashkenazi and Sephardi Levites are genetically dissimilar, in contrast to what was found for Ashkenazi and Sephardic Cohanim (Hammer et al., 2009).

A very recent study (Tofanelli et al., 2014) of the haplotype motifs of Levites and Cohanim Jewish Priestly lineages has, however, found that these supposed markers of Jewish ancestry can lead to ambiguous results since they are not identical by descent. These motifs were observed in independent lineages from different ethnic, cultural and geographic groups, probably due to multiple founder events, recombination and admixture of the Jewish genetic pool in the course of their history (Tofanelli et al., 2014).

**FIGURE 1:**
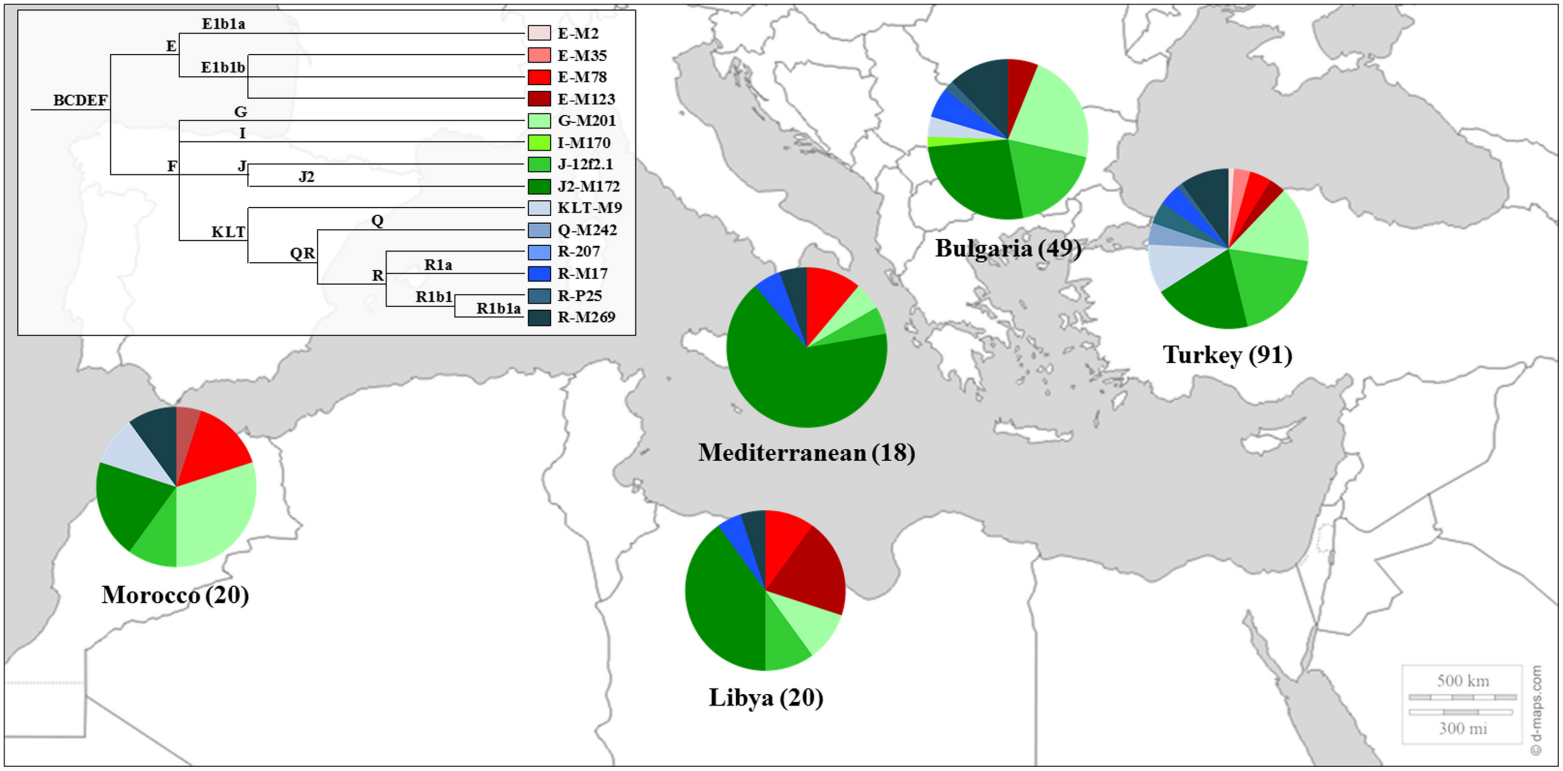
**Y Chromosome haplogroup distributions of Sephardic Jewish populations.** Sectors in pie charts are proportional to haplogroup frequency. Number of total individuals (n) are in brackets for each population. Jewish samples are from the works of Shen et al. (2004) and Adams et al. (2008).

Sephardic Jews were also included in studies of the Y chromosome phylogeography (Semino et al., 2004), in the genetic affinities between Jews and other populations from the Middle East (Shen et al., 2004; Oefner et al., 2013) and in the construction of the genetic landscape of the Iberian Peninsula (Adams et al., 2008). The genetic profile of the Sephardic Jews can be seen in **Figure 1**.

Several studies refer to the putative contribution of Sephardic Jews to the Y chromosome genetic pool of particular geographic regions, like the Azores and Madeira islands (Goncalves et al., 2005; Pacheco et al., 2005) and also to the New-World, such as Brazil (Carvalho-Silva et al., 2001) or New Mexico and southern Colorado (Sutton et al., 2006), where many populations settled, including migrants from Portugal/Iberia.

### Y CHROMOSOME IN SEPHARDIC PORTUGUESE JEWS

The profile of male lineages in Portugal was drafted in a study comprising 663 male samples from the 18 administrative districts of Portugal and a typical western European composition was demonstrated by the high frequencies of haplogroups R1b1a-M269 (57.7%), I-M170 (6.1%), G-M201 (5.5%), and E1b1b-M81 (5.6%), as well as a Middle Eastern influence, denoted by the presence of J-12f2.1 lineage (10.4%; Beleza et al., 2006). Possible Sephardic contributions to this genetic pool were also addressed in some reports (Goncalves et al., 2005; Pacheco et al., 2005) but very little was known about the Portuguese Jews, even though, in a large scale study of the Iberian genetic diversity, very few Jewish male samples from Belmonte were analyzed (Adams et al., 2008).

These samples were included in a larger group of self-defined Sephardic Jewish males not only from the Iberia Peninsula but also from other countries that received Jewish exiles after the decrees of expulsion in the 15th century. This group of self-defined Sephardic Jews was treated as a single group, therefore the inference of a genetic profile for the Portuguese Jews was not possible from the published data. In the following, we will focus on the results obtained exclusively for the 16 Jewish samples of Belmonte, obtained upon request to the authors (Adams et al., 2008).

**FIGURE 2:**
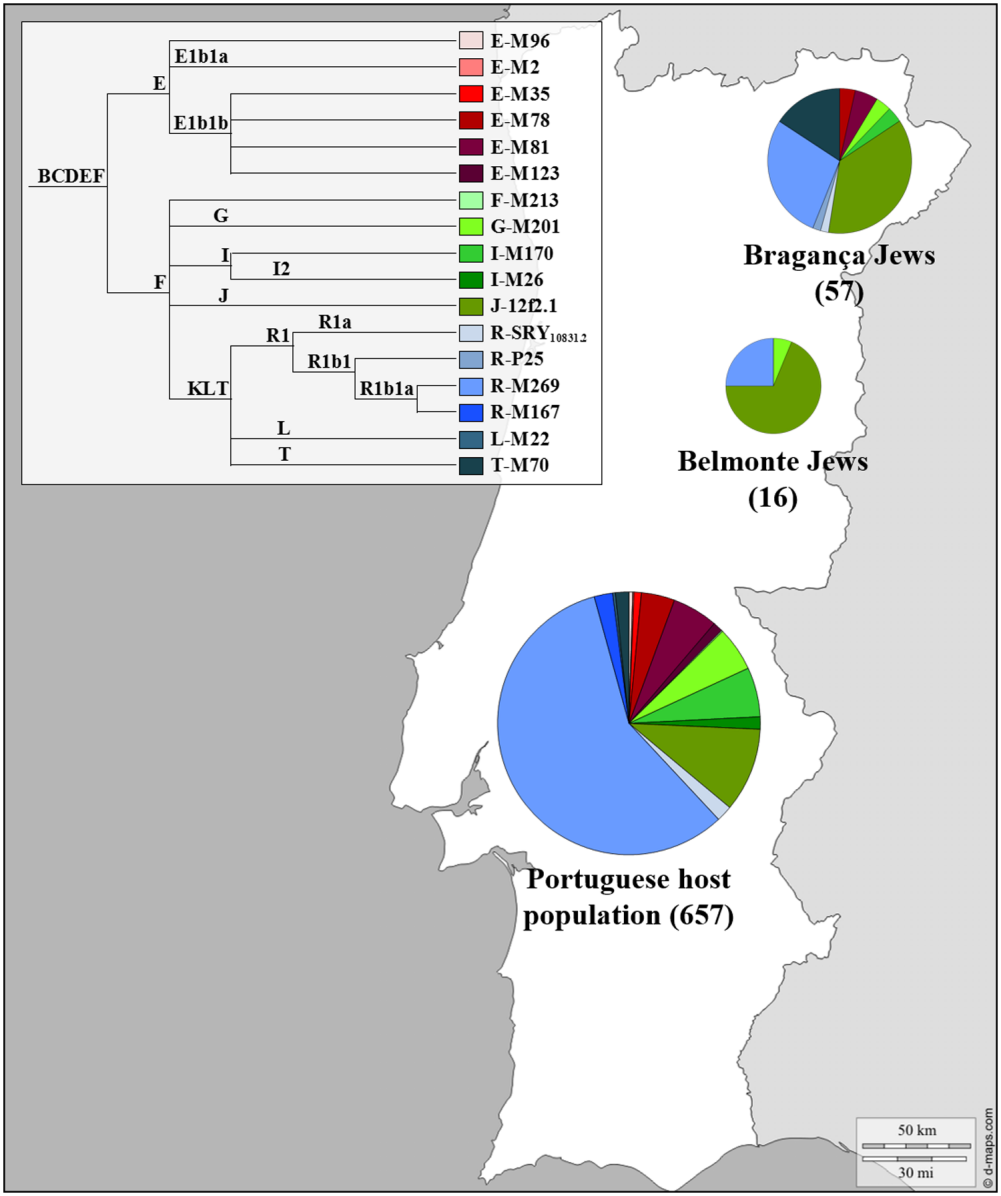
**Y Chromosome haplogroup distributions of the Portuguese Sephardic Jews and non-Jewish population.** Sectors in pie charts are proportional to haplogroup frequency. Number of total individuals (n) are in brackets for each population. Jewish samples (Belmonte and Bragança) are from the works of Adams et al. (2008) and Nogueiro et al. (2010), respectively, and the Portuguese host population from Beleza et al. (2006).

The genetic profile of the Portuguese Jewish and non-Jewish male lineages can be seen in **Figure 2**. The Y chromosome SNPs analyzed allowed the definition of just three different lineages in Belmonte Jews: eleven individuals were classified as J-12f2.1, four as R1b1a-M269 and one as G-M201, with a frequency of 68.8, 25 and 6.2% respectively. The analyses of the STRs DYS19, DYS388, DYS389I-II, DYS390, DYS391, DYS392, DYS393, DYS438, DYS439, DYS385a, and DYS385b revealed a total of only four distinct haplotypes. In the R1b1a-M269 haplogroup two different haplotypes were detected, diverging one from the other by one mutation step (DYS389II), inside the J-12f2.1 haplogroup all the eleven individuals presented exactly the same haplotype, reflecting very low levels of genetic diversity among this Jewish community.

A completely different picture of the Portuguese male Jewish lineages was, however, brought to light when the descendants of the crypto-Jews from Bragança district were analyzed (Nogueiro et al., 2010). In this study, 57 unrelated self-designated Jewish males from the Northeast Portugal (Bragança, Argozelo, Carção, Mogadouro, and Vilarinho dos Galegos) were selected, using a combination of geographic, religious ethno-historical and affiliation criteria.

The SNPs typed allowed the discrimination of 10 different haplogroups and the analysis of the Y-STR loci revealed 41 different haplotypes. The most frequent haplogroups found were R1b1a-M269, J-12f2.1, and T-M70, adding up to 80.7% of the total sample (**Figure 2**).

The effect of genetic drift in an isolated, small sized population could explain the high frequency found in Bragança for lineages typically predominant in other Jewish populations, such as J-12f2.1 (36.8%) and T-M70 (15.8%). However, the high haplogroup diversity combined with the high (intra-haplogroup) haplotypic diversity are extremely surprising, as they show exactly the opposite of what is expected, namely a deep genetic diversity loss. Although inbreeding practices were sustained by the Portuguese crypto-Jewish communities, in the light of the obtained results it seems that its effects were less pronounced in the Bragança district compared to Belmonte, due probably to complex mating strategies and/or a very heterogeneous genetic pool in their origin.

Haplogroup R1b1a-M269, representative of Western Europe, is the most common lineage found in the Portuguese general population (57.7%; Beleza et al., 2006) while in the Jewish population it does not go beyond 28.1%. In contrast, haplogroup J-12f2.1, typical in other Jewish populations, appeared with a frequency of 36.8%, contrasting with the low frequency found in the Portuguese population (10.4%; Beleza et al., 2006). The same happened with haplogroup T-M70 (15.8%), which is quite rare among the non-Jewish Portuguese lineages (1.6%; Beleza et al., 2006).

Haplogroup R1b1a-M269, emerges as the most frequent lineage in European individuals. Its distribution displays an increasing gradient moving from east to west (Semino et al., 2000; Bosch et al., 2001; Cruciani et al., 2002; Flores et al., 2004; Brion et al., 2005; Moore et al., 2006), being the most frequent haplogroup in the Iberian Peninsula. Lineage R1b1a-M269 was associated with the expanding Neolithic movements from the Near East to the western fringe of Europe, although this is still a matter of debate (Balaresque et al., 2010; Busby et al., 2011).This haplogroup was absent in Jewish population studies until the report of Adams et al. (2008).

The high frequency of haplogroup R1b1a-M269 found in both groups of Portuguese Jews could result from admixture with the Portuguese/Iberian population, and/or from introgression before their entry into the Iberian Peninsula. Pairwise *R*_ST_ genetic distances between R1b1a-M269 Jewish haplotypes from Bragança district and those from Portugal (Beleza et al., 2006) and Turkey (Cinnioǧlu et al., 2004), were analyzed by Nogueiro et al. (2010) to verify the contributions of Western Europe versus Near East to the frequency of the Portuguese Jewish R1b1a-M269. A lower genetic distance between the Portuguese Jewish and non-Jewish R1b1a-M269 haplotypes than between these two samples and the one from Turkey was detected. Thus, an important Western European R1b1a-M269 introgression into the Portuguese Sephardic Jews, most probably after their arrival in Iberia, is the most plausible scenario (Nogueiro et al., 2010).

No exact matches, or total identity in all markers, were found between R1b1a-M269 haplotypes from Bragança district and those from Belmonte Jews. However, only two mutation steps distinguish each of the two Belmonte haplotypes (which differ by just one mutation step) from the one found in Bragança.

Haplogroup J-12f2.1 has a Middle Eastern origin and includes two groups, the J1-M267 and the J2-M172. Lineage J2-M172 is more common and is widely spread over Europe, particularly in the Mediterranean basin (Semino et al., 2004). Haplogroup J-12f2.1 presents a decreasing gradient from its origin toward Europe and is associated with the demic diffusion of the Neolithic farmers (Underhill et al., 2001; Semino et al., 2004) and also to more recent events, such as the Phoenician maritime migrations along the Mediterranean (Hammer et al., 2000; Di Giacomo et al., 2004; Zalloua et al., 2008).

This haplogroup is referred to as being predominant in diverse referenced Jewish populations (Hammer et al., 1997, 2000; Nebel et al., 2001; Adams et al., 2008), reaching in Sephardic Jews, frequencies above 40% (Semino et al., 2004; Adams et al., 2008). While in Portugal it accounts for 3.4% of J-12f2.1 and 7% of J2-M172 lineages (Beleza et al., 2005) in the Portuguese Jews, it reached values of 68.2% for J-12f2.1 in Belmonte, 12.3% and 24.5% for J1-M267 and J2-M172 respectively, in the Bragança district.

The high frequencies of J-12f2.1 haplogroup found in both groups of Portuguese Jews, compared to the non-Jewish Portuguese host population, could therefore represent part of the genetic pool of the ancestral Sephardic population that established the first Jewish settlements in Portugal. For this lineage, again, no exact matches were found between the haplotypes of Belmonte and Bragança district. Three mutational steps apart, one haplotype of Belmonte matches five individuals from Bragança, Carção, Argozelo, and Vilarinho dos Galegos.

The presence of the mutation M70 defines haplogroup T. Its origin is attributed to the Middle East (Underhill et al., 2001) and from there it spread along the Mediterranean and East Africa. It is a rather rare haplogroup, displaying a global frequency of around 1% (King et al., 2007), but nonetheless it is found at quite high frequencies in Sephardic Levites (23%) and Sephardic Israelis (13%; Behar et al., 2004).

In Portugal it accounts for just 1.6% (Beleza et al., 2006) but reaches 15.8% in Bragança district Jews, being absent in the Belmonte samples. This lineage probably represents a relic of the original Sephardic male genetic pool, since it appears with similar frequencies in Israeli Sephardic Jews, but is quite rare in the Mediterranean coast and in Iberia.

Several other haplogroups were detected in the NE Portuguese Jews with residual frequencies, namely E1b1b-M78 with 3.5%, E1b1b-M81 with 5.2%, I-M170with 3.5% R1b1-P25 with 1.8%, R1a-SRY_10831.2_ with 1.8%, and G-M201 with 3.5%.

G-M201 was also detected in Belmonte (6.2%) at about the same frequency as in the non-Jewish Portuguese population. Adams et al. (2008) suggested that this haplogroup could reveal an introgression of Sephardic Jews into the Iberian population. However, the estimated age for this lineage in Portugal (Beleza et al., 2005) is consistent with its introduction during the Neolithic and the results of relative frequencies and STR variance inside this lineage from Adams et al. (2008) does not allow the definition of the gene flow direction.

### mtDNA STUDIES IN SEPHARDIC POPULATIONS

The first work dealing with the maternal (mtDNA) lineages in Sephardic populations dates from 1986 (Bonne-Tamir et al., 1986). The authors studied mtDNA variation patterns in a sample of 81 Arab and Jewish Israelis, including three individuals of Sephardic origin and a possible existence of group-specific mtDNA fragment patterns was speculated. Shortly after, in 1991, a complementary work (Tikochinski et al., 1991) increased the available data, with 39 Jewish individuals including 18 Sephardic samples, mainly of Moroccan origin. Twenty-one distinct maternal linages were identified but no estimation of the introgression degree from the host population was performed.

Later (Thomas et al., 2002), HVRI segments of the mtDNA Control Region (CR) were sequenced in 615 Jewish individuals belonging to nine geographically separated groups. The work included a large sample of 115 Moroccan Jews, a community that, as previously stated, received Iberian Jews after their expulsion. The HVRI frequencies for the Moroccan Jews showed a high prevalence (27%) of sequences presenting no differences to the revised Cambridge Reference Sequence (rCRS), and so, included in the H haplogroup, which although ubiquitous in Europe has a significantly higher prevalence in the Iberian Peninsula.

**FIGURE 3:**
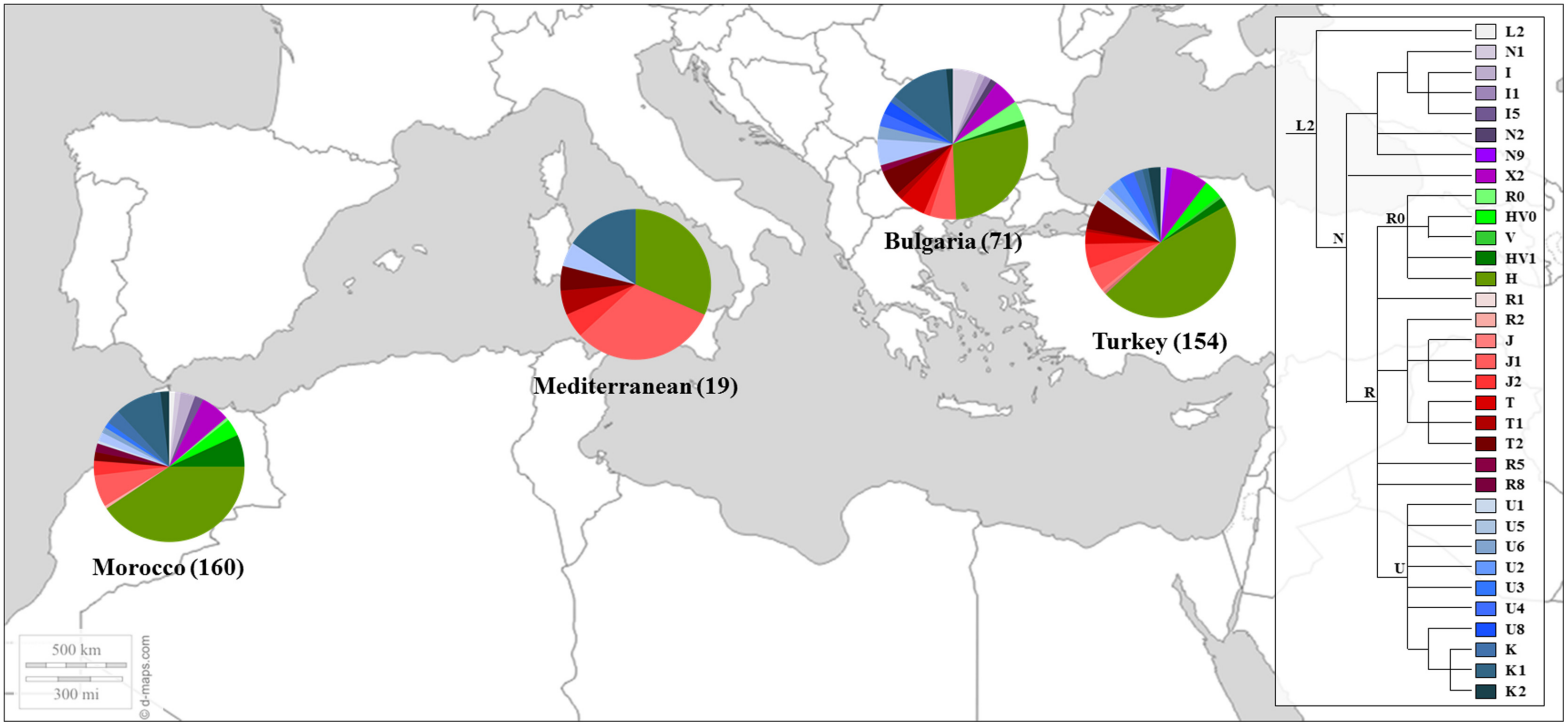
**Mitochondrial haplogroup distributions of the Sephardic Jewish populations.** Sectors in pie charts are proportional to haplogroup frequency. Number of total individuals (n) are in brackets for each population. Jewish samples are from the works of Picornell et al. (2006) and Behar et al. (2008).

Similar results were found in later works (Picornell et al., 2006; Behar et al., 2008), including samples of Sephardic Jewish communities. In the work of Picornell et al. (2006) the HVRI and HVRII were analyzed in 31 individuals from Turkey and 12 from Morocco while Behar et al. (2008) studied a larger fragment of the mtDNA control region (16,024-300) together with diagnostic positions of the coding region in 149 Moroccans and 213 individuals from different Sephardic communities (e.g., Bulgarian and Turks). These communities also presented a high proportions of mtDNA haplogroup H. Moreover, a remarkable west Mediterranean imprint among the Turkic Jewish sample was observed (Behar et al., 2008). The genetic profile of the referred Jewish’s populations is shown in **Figure 3**.

The detection of traces of Sephardic Jewish presence was addressed in several populations through the analysis of their possible contribution to the genetic background of the host populations. Such contribution was investigated in regions like the Portuguese north Atlantic archipelagos of Madeira and Azores (Brehm et al., 2003; Santos et al., 2003, 2010) or the Spanish population of Pasiegos, an isolate from the northern region of Cantabria (Maca-Meyer et al., 2003). HVRI was also analyzed in a sample of 45 *Chuetas* (Picornell et al., 2005) showing a high frequency (23% the most prevalent) of Middle Eastern haplogroup R0a. This pattern of mtDNA diversity, showing haplogroups from the Middle East, was associated with female-specific founder events and has been described in various Jewish communities (Thomas et al., 2002; Behar et al., 2008).

Such contribution was also analyzed in the New World populations, since Jewish migrations after the expulsion are well documented (Barnavi et al., 1992; Bacon, 2011). One of the first works was produced by Carvajal-Carmona et al. (2000), comprising 80 Colombian individuals from the Antioquia province. Only restriction diagnostic sites of the four major founder Native American mitochondrial haplogroups (A–D) were examined, and therefore, due to the low resolution power, it was not possible to carry out further insights on the ancestry of the non-Amerindian lineages detected (∼10%).

Two Latin American populations, (inhabitants of Loja province in Sothern Ecuador and the Hispanos community from San Luis Valley of Colorado in Southwest US), with a presumable Sephardic Jewish ancestry were analyzed by Velez et al. (2012). The selection of such communities was done based on the occurrence of two mutations previously associated to genetic conditions described in different Jewish communities (Ostrer, 2001). HVRI mtDNA sequence variation and RLFP analysis of diagnostic haplogroup positions of the coding region from 53 individuals revealed, as in the previous work, a high prevalence of the founder Native American lineages (∼93%).

Complementary strategies have also been used to study this possible Sephardic contribution in a more recent work (Bedford, 2011). The analysis of CR shared haplotypes in T2e haplogroup, using different public mtDNA databases, was reported. The authors focused the analysis in a specific CR motif (combination of certain variations) initially named as T2e5, which can be currently located into the T2e1a1a1 clade, according to the updated mtDNA phylogeny in PhyloTree built 16 (van Oven and Kayser, 2009). The complete mtDNA genome from a Turkish Sephardic individual belonging to this rare clade was also sequenced. The authors observed the motif in twelve samples including Sephardic descendants from Turkey and Bulgaria, individuals from North American regions (Northern Mexico and South USA, places known for receiving Spanish *Conversos*), and samples from Portugal and Brazil, also consistent with a speculated Sephardic ancestry.

Further insights regarding the Sephardic signature inside T2e haplogroup and the genetic affinities of the T2e Northern Mexican samples were presented later (Bedford et al., 2013). The mitogenomes analyzed allowed to clarify the phylogeny of the Sephardic branches T2e1a and T2e1b. Indeed, the perfect match between complete sequences of Mexican individuals belonging to T2e1a clade and those from Turkish/Bulgarian Sephardic individuals, provided genetic evidence for a Sephardic origin of this lineage.

### mtDNA IN SEPHARDIC PORTUGUESE JEWS

In parallel with the analysis of the male counterpart (Y chromosome), the mtDNA variation in Portugal was used to investigate the maternal heritage in the current Portuguese genetic landscape. The first detailed report on the Portuguese mtDNA, was done by Pereira et al. (2000), where HVRI and HVRII of 549 samples from North, Central and South Portugal were typed. The Portuguese population presented a typically Western European mtDNA composition with the distinction of harboring higher frequencies of North and Sub-Saharan African specific lineages (haplogroups L1-3 and U6). Similar results were found in a later work (Gonzalez et al., 2003), with the sequencing of the control region HVRI in 299 Portuguese samples. Specific areas of Portugal were analyzed in detail, due to their distinctive geographic and demographic characteristics: Azores and Madeira islands have a recent settlement history and played an important role in the modern slave trade from Africa to the New World, which is reflected in the significant presence of sub Saharan lineages (Brehm et al., 2003; Santos et al., 2003, 2010); The populations of Coruche, Pias, and Alcacer do Sal, were analyzed due to a recent malaria endemicity with different mtDNA compositions (Pereira et al., 2010); The Northeast population of Miranda do Douro near the Portuguese-Spanish border (Mairal et al., 2013) was also recently studied for the singularity of their Mirandese language and a diversity decrease of mtDNA lineages was found for this small-sized and isolated population.

**FIGURE 4:**
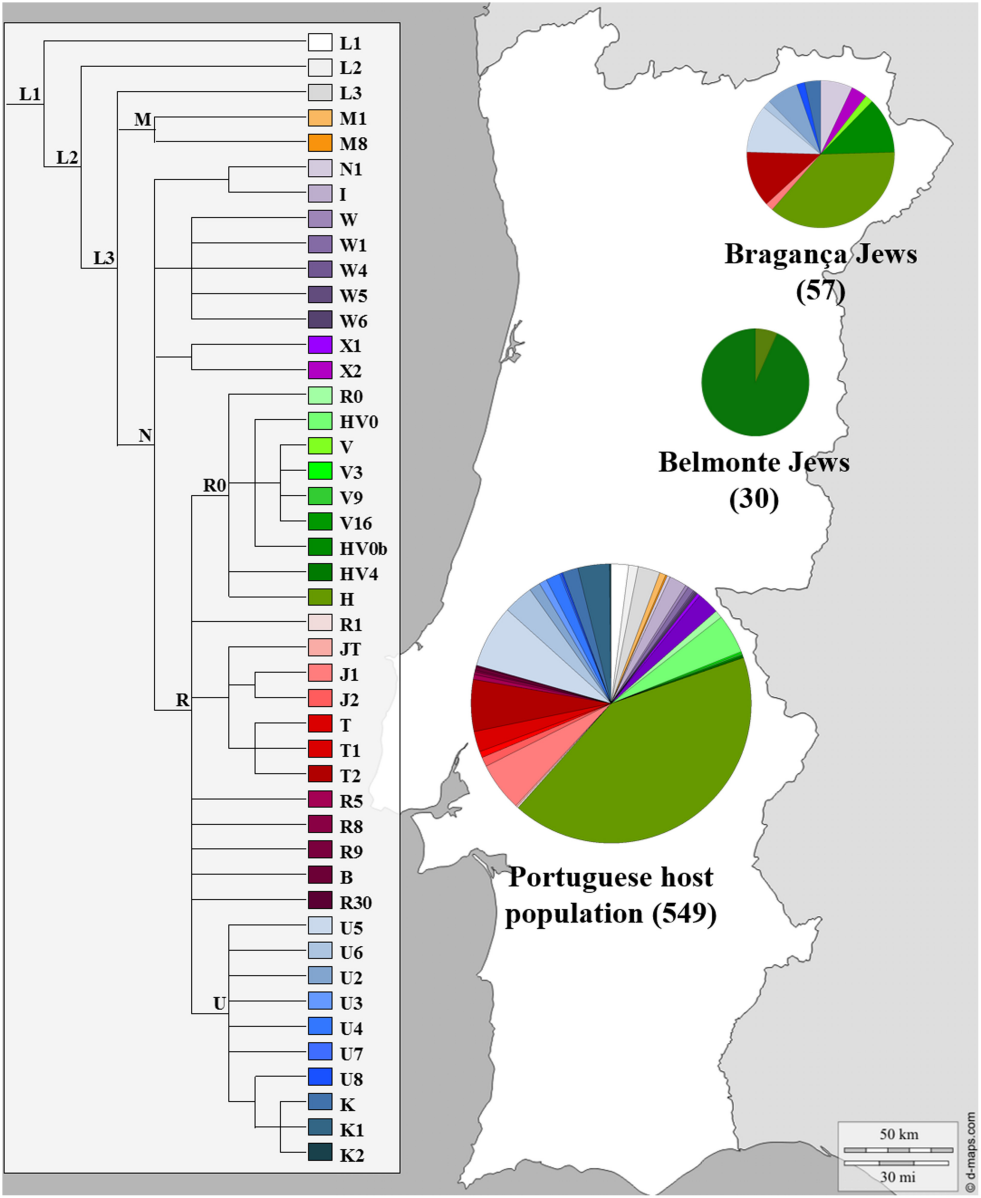
**Mitochondrial haplogroup distributions of the Portuguese Sephardic Jews and non-Jewish population.** Sectors in pie charts are proportional to haplogroup frequency. Number of total individuals (n) are in brackets for each population. Jewish samples (Belmonte and Bragança) are from the works of Behar et al. (2008) and Nogueiro et al. (2014), respectively, and the Portuguese host population from Pereira et al. (2000).

The possible contribution of Sephardic lineages to the female Portuguese genetic pool was investigated as previously stated (Brehm et al., 2003; Santos et al., 2003, 2010) nevertheless only three published reports on mtDNA variation actually included Portuguese Sephardic Jewish descendants (Behar et al., 2008; Teixeira et al., 2011; Nogueiro et al., 2014). Behar et al. (2008) analyzed mtDNA CR along with diagnostic positions of the coding region in 30 Jewish individuals from the community of Belmonte municipality in Central Portugal, while the most recent one, studied the complete mtDNA molecule in 57 self-designated Jewish descendants, sampled in several locations in Bragança district (NE Portugal): Bragança, Argozelo, Carção, Mogadouro and Vilarinho dos Galegos, (Nogueiro et al., 2014). The genetic profile of the Portuguese Jews and the Portuguese non-Jewish female lineages can be seen in **Figure 4**.

The Belmonte community presented a very low diversity, with only two lineages detected, and all samples inside each haplogroup presented the same haplotype. The distribution of the haplogroups is uneven, since one of them, HV0b haplogroup, stands out with a frequency of 93.3%. On the other hand, the Bragança Jewish sample presented a much higher haplogroup diversity (32 haplogroups) as well as intra-haplogroup diversity (45 haplotypes). The haplogroups that stand out in the Bragança Jewish sample, when compared with the Portuguese host population, were: HV0b, N1, T2 and U2, adding up to 38.6%.

The single mtDNA lineage shared between the two analyzed Portuguese Jewish series, Belmonte and Bragança, is HV0b. The expansion of the haplogroup HV0, including the haplogroup V, had been proposed to have an Iberian origin, after the last glacial maximum, and nowadays can be also found in North African populations. However, little is known about the distribution of the HV0b clade. According to the CR mutations, the specific defining combination variants (16298C, 72C, 195C, 198T and 263G) can be found at EMPOP forensic database in nine individuals, including one from Northern Africa; five from Central and North America; two from Central Europe; and one from Southern Portugal. Moreover, a recent paper focused on mtDNA variation in Andalusia (Southern Spain), (Hernández et al., 2014) reported samples that can be included inside this haplogroup but with additional variations: one sample from Granada (16093C, 16293G, 16298C, 16519C, 72C, 195C, 198T, and 263G) and two from Huelva (16153A, 16298C, 72C, 195C, 198T, and 263G), the latter being also found in ethnic Portuguese Roma (Mendizabal et al., 2011). This scenario is compatible with its origin in the Iberian Peninsula and with an early introgression into the Iberian Jewry gene pool from its host population, as suggested by Behar et al. (2008).

Looking deeper into the variability of the HV0b clade through the comparison of complete mtDNA sequences from Bragança Jews (seven), Belmonte Jews (one) and GeneBank (two), reveals a shared common private variant in the coding region (8520G) of the Portuguese Jewish samples. Moreover, the Bragança samples cluster together, sharing a variant (10644A), which seems to have arisen locally. Although new complete mtDNA sequences are needed for a better understanding of this particular clade, the results available so far support the hypothesis that at least the HV0b-8520G haplotype is a Sephardic Jewish founding lineage.

Another interesting clade that deserves further attention is T2e1. This clade was described as one of the founder lineages of the Bulgarian Sephardic community (Behar et al., 2008), and as referred to previously, is found among populations from Northern Mexico and Southern USA, being interpreted as a Sephardic signature (Bedford, 2011; Bedford et al., 2013). A Near East origin for haplogroup T was proposed with a posterior expansion into Europe before the Neolithic. The distribution of some sub-clades, including the T2e lineage, was associated with posterior European indigenous dispersion events (Pala et al., 2012). Three Bragança Jewish samples belong to T2e1 clade, two of which can be further classified as T2e1a and one as T2e1b, according to coding region variations (2308G and 9181G, respectively; van Oven and Kayser, 2009). Inside T2e1a branch the Bragança Jewish samples can be further classified into T2e1a1 sub-branch, based on two CR variations (back-mutation at position 41 and 16192T) and as T2e1a1a based on the presence of the CR variation (16114T). Inside T2e1a1a, Bragança Jewish sequences cluster together with three samples, one Sephardic sample from Turkey, and two other from Northern Mexico and Southern USA (Bedford et al., 2013). Moreover, the Braganca Jews present two further distinct coding region variants (13135A and 7133T). As stated by Bedford et al. (2013), the ancestry of the analyzed Northern American samples are consistent with an Iberian Sephardic origin. Apart from the Bragança T2e1b sample, this branch contains 10 other complete mtDNA sequences. Eight of them, with widespread geographic European origin, are of Jewish ethnicity, either Sephardic (Netherlands, Romania and Bulgaria) or Ashkenazi (Poland, Czech Republic, and Lithuania; Bedford et al., 2013).

Similar results regarding shared haplotypes between samples from Sephardic and Ashkenazi origin were found inside U2e1a1 sub-clade. All four U2e1a1 samples from Bragança Jews share the same haplotype with a Jewish Ashkenazi sample from Moldova (Behar et al., 2012), presenting two private CR variations (8014G and 13708A).

This pattern of shared haplotypes between Sephardim and Ashkenazim samples, firstly described by Bedford et al. (2013), could represent two possible scenarios (Nogueiro et al., 2014): the defining variants could have arisen before the separation between the two Jewish communities; or it may have resulted from a recent introgression of Sephardic lineages into the Ashkenazi gene pool. Further genetic data will help to clarify this issue, but it is possible to add non-genetic evidence for the second hypothesis, since marriages between members of the two communities have been recorded (Roth, 1979; Elvira, 2007), the descendants having been assimilated into the Ashkenazi community.

## FINAL REMARKS

In conclusion, the demographic processes underlying the genetic pool of the Portuguese Crypto-Jews descendants studied so far, are much more complex than would be expected under the classical model of extreme inbreeding and drift, with consequent loss of genetic diversity. The contrasting patterns observed in Bragança community and Belmonte are enough to sustain that whatever the results of future studies, no simple and uniform evolutionary model will accommodate the sharp heterogeneity already observed.

Notwithstanding this difference, both groups display a genetic pool clearly showing contributions of European and Near Eastern lineages, in accordance with a significant persistence of a Jewish heritage, translated in a conscience of belonging to a distinctive community. This ancestry was detected within both male and female lineages, indicating that introgression from and admixture with the host population does seem to have been significantly gender biased.

Moreover, the high genetic diversity found in Bragança demonstrates that there was neither a low number of founder lineages, nor a significant reduction of effective population size as indeed occurred in Belmonte. It remains to be explained how this resistance to genetic erosion, as expected in endogamous, small sized populations, was achieved, that is to say, what mating strategies were undertaken by these communities which ensured a steady gene flow between them, counteracting the expected inbreeding.

New data from recombining genetic markers in the line of Behar et al. (2010), as well as from classical genealogical studies, will surely contribute decisively to explain how this was achieved. At any rate, the DNA evidence gathered so far adds a new facet to the already recognized astonishing cultural resistance of these communities: not only they have kept a sense of belonging throughout centuries of persecution but they also succeeded in maintaining a genetic heritage of their own.

## Conflict of Interest Statement

The authors declare that the research was conducted in the absence of any commercial or financial relationships that could be construed as a potential conflict of interest.
